# A construct divided: prosocial behavior as helping, sharing, and comforting subtypes

**DOI:** 10.3389/fpsyg.2014.00958

**Published:** 2014-09-02

**Authors:** Kristen A. Dunfield

**Affiliations:** Department of Psychology, Center for Research in Human Development, Concordia UniversityMontreal, QC, Canada

**Keywords:** prosocial behavior, social-cognitive development, emotional development

## Abstract

The development and maintenance of prosocial, other-oriented behaviors has been of considerable recent interest. Though it is clear that prosocial behaviors emerge early and play a uniquely important role in the social lives of humans, there is less consensus regarding the mechanisms that underlie and maintain these fundamental acts. The goal of this paper is to clarify inconsistencies in our understanding of the early emergence and development of prosocial behavior by proposing a taxonomy of prosocial behavior anchored in the social-cognitive constraints that underlie the ability to act on behalf of others. I will argue that within the general domain of prosocial behavior, other-oriented actions can be categorized into three distinct types (helping, sharing, and comforting) that reflect responses to three distinct negative states (instrumental need, unmet material desire, and emotional distress). In support of this proposal, I will demonstrate that the three varieties of prosocial behavior show unique ages of onset, uncorrelated patterns of production, and distinct patterns of individual differences. Importantly, by differentiating specific varieties of prosocial behavior within the general category, we can begin to explain inconsistencies in the past literature and provide a framework for directing future research into the ontogenetic origins of these essential social behaviors.

Humans have a number of exceptional abilities, one of which is our pervasive, obligatory sociality (Brewer and Caporael, 2006). Not only do humans regularly act *with* others, we also often act on *behalf* of others (e.g., Tomasello, 2009). Importantly, this other-oriented tendency has long been recognized as an intriguing explanatory puzzle. Specifically, from a strict Darwinian “survival of the fittest” perspective, behaviors that benefit another at a cost to one’s self should not exist, largely because the temptation to, and benefits of, cheating are simply too high (e.g., Darwin, 1859; Dawkins, 1989). Yet, despite the explanatory challenges, other-oriented acts do exist and appear to be an essential (Tomasello, 2009), automatic (Zaki and Mitchell, 2013), universal (e.g., Henrich et al., 2005; Callaghan et al., 2011), and relatively unique (e.g., Warneken and Tomasello, 2009; Silk and House, 2011) part of human social life.

The ability and willingness to engage in prosocial behavior appears to have important implications for well-being at the individual (e.g., Crick, 1996; Sallquist et al., 2012), group (Anderson and Kilduff, 2009), and societal (Zak, 2008; Tomasello, 2009; Pinker, 2011) level of analysis. Due in part to their intriguing theoretical constraints (Hamilton, 1964; Trivers, 1971), and in part to their widespread social implications (Tomasello, 2009; Pinker, 2011), other-oriented behaviors have captured the curiosity of scholars from a variety of disciplines (e.g., Bowles and Gintis, 2011; Wilson, 2012; Bloom, 2013; Greene, 2013). This diverse interest has resulted in a large body of literature examining the factors that support the emergence and maintenance of these essential social acts across both phylogeny (Warneken and Melis, 2012) and ontogeny (Eisenberg et al., in press). Yet, instead of providing clarity and insight, these diverse research programs have brought to light a number of challenges and controversies in our current understanding of prosocial development. For example, different measures of prosocial behavior are often uncorrelated (e.g., Hay and Cook, 2007; Dunfield and Kuhlmeier, 2013), early prosociality often correlates with aggressive tendencies (e.g., Hay, 2006), and children regularly ignore or exacerbate the distress of others (Dunn, 1988).

The goal of this paper is to shed light on some of these explanatory challenges by considering prosocial behavior from the perspective of social-cognitive development. Specifically, I will propose that within the general domain of prosocial behavior there are three distinct varieties of responses that can be differentiated based on their unique underlying social-cognitive constraints. Then, I will provide evidence for the utility of this distinction by demonstrating that these behaviors show dissociable developmental trajectories and distinct associations with individual difference factors early in life. As this paper is intended to organize and direct research into the emergence and early development of prosocial behavior, the focus will be on the rapidly growing body of literature examining prosociality from infancy through early childhood.

## DEFINING PROSOCIAL BEHAVIOR

There are many ways to act on behalf of others. Typically we apply the term “prosocial” to any behavior that is intended to benefit another (e.g., Eisenberg, 1986). Utilizing this broad definition, numerous studies have demonstrated that humans appear exceptional in their ability to respond to a diversity of needs (Svetlova et al., 2010; Dunfield et al., 2011; Dunfield and Kuhlmeier, 2013), very early in development (Zahn-Waxler et al., 1992; Warneken and Tomasello, 2006). Though we have made great strides in documenting the myriad of prosocial behaviors that children can produce, we still have much to learn about the mechanisms that underlie and support these fundamental acts (see Radke-Yarrow et al., 1983 for a historical, yet relevant, perspective on similar issues).

While many have *hypothesized* supporting mechanisms such as socialization (Hastings et al., 2007), cognitive development (e.g., perspective taking, Hoffman, 1982; Underwood and Moore, 1982), or underlying individual differences (e.g., prosocial personality, Eisenberg et al., 1999; genetic underpinnings, Knafo and Israel, 2009), these claims have been difficult to evaluate. A historical tendency to employ a broad definition of prosocial behavior and naturalistic or observational designs (Schroeder et al., 1995; Eisenberg et al., 2006) has resulted in limited consistency charting the age of emergence (e.g., Zahn-Waxler et al., 1992), developmental trajectories (e.g., Radke-Yarrow et al., 1983), behavioral correlates (e.g., Eisen-berg and Hand, 1979), and individual differences associated with production of other-oriented acts. Indeed, treating all prosocial behaviors as similar “kinds” has resulted in much difficulty developing coherent theories regarding developmental mechanisms (see Radke-Yarrow et al., 1976; Eisen-berg and Hand, 1979; Zahn-Waxler et al., 1992 for notable exceptions).

Part of the explanatory difficulty may result from a tendency to consider prosocial development from either an individual difference *or* developmental universal perspective (e.g., Nichols et al., 2009). Individual difference (dispositional) accounts attempt to explain variability in the propensity to act prosocially by examining stable individual difference factors such as emotion regulation, contentiousness, or inhibitory control. Though there is support for this perspective (e.g., Eisenberg et al., 1999), the pattern of relations is not always consistent. For example, though spontaneous prosocial behavior in preschool predicts other- and self-reported prosocial behavior in early adulthood, compliant and low-cost helping did not. Importantly, the mechanism underlying these variable relations is not always clear. One possibility is that that methodological limitations associated with assessing motivation in infancy and early childhood are limiting our ability to identify the relevant relations (Thompson and Newton, 2013). Alternatively, it’s possible that the variability reflects the fact that prosocial motivation is diverse (e.g., Eisenberg et al., 1991; Paulus, 2014).

Developmental accounts, on the other hand, typically examine how the acquisition of various universal cognitive skills, such as mental state understanding, affects the production of prosocial behavior. These accounts seek to explain similarities across individuals in the development of prosocial behaviors by first identifying universal milestones in the development of prosocial behavior, then identifying the underlying social cognitive correlates. These two varieties of accounts are *not* mutually exclusive, and there is reason to think that both dispositional and developmental factors work in concert to support the production of prosocial behavior (e.g., Nichols et al., 2009). Specifically, it has been suggested that prosocial behavior can be considered both a general, superordinate category that contains a variety of distinct responses (i.e., a prosocial disposition), but also a construct that gains breath and complexity with development (i.e., a developmental universal; Thompson and Newton, 2013). By taking a developmental universal perspective, the current paper seeks to clarify the variety of ways humans act prosocially with the hope that by clarifying the various manifestations of prosocial behavior and their unique constraints, we can gain better insight into the interplay between developmental universals and individual differences in the production of prosocial behavior.

### A DEVELOPMENTAL UNIVERSAL PERSPECTIVE

One way that we may address and overcome some of the current explanatory limitations is by clarifying the variety of ways that humans act prosocially. The current proposal builds off of existing categorizations that acknowledge heterogeneity in the various manifestations of prosocial behavior and recognize an important role for social cognitive development in the production of early prosocial acts (e.g., Hay and Cook, 2007; Warneken and Tomasello, 2009; Brownell et al., 2013b). However, the current proposal differs from previous categorizations in the emphasis placed on the primary mental state evaluation that the individual is required to make when determining whether and how to aid another.

Regardless of what the prosocial actor does or why, the central characteristic underlying the dissociation of the various prosocial responses is the primary negative state that the actor is recognizing and responding to. For example, effectively alleviating distress in a crying individual whose stomach is rumbling would depend on whether the affective response is a cause or consequence of the hunger. An individual who is so hungry they become upset requires a very different intervention than an individual who is so upset they lose their appetite. In the first case, reducing hunger by offering food will alleviate the emotional distress; in the second case, reducing emotional distress by offering social support will (eventually) alleviate the hunger (by allowing an anxious appetite to return). This fit between the initial eliciting event and the appropriate/effective intervention is a *fundamental* but commonly overlooked part of engaging in prosocial behavior.

There is growing consensus that understanding prosocial behavior will require a multidimensional approach that considers the variety of distinct mechanisms that may lead to different prosocial responses (e.g., Hay and Cook, 2007; Dunfield and Kuhlmeier, 2013; Thompson and Newton, 2013; Paulus, 2014). Categorizing varieties of prosocial behavior based on the negative state they respond to seems to be a fruitful conceptualization because considerable past research has demonstrated that from very early in development humans automatically identify others’ mental states (including goals, beliefs, and desires) and then use these evaluations to understand and predict others’ behavior (e.g., Frith, 2012). This tendency to automatically attribute and share mental states is thought to play an integral role in human social interactions, so much so that it has been argued that a primary function of explicit metacognition is to enhance social relations and support fruitful group interactions (e.g., Tomasello et al., 2005; Tomasello, 2009; Frith, 2012).

Consistent with this claim, previous studies have found that as children’s social-cognitive capacities mature so does their ability to work with (Brownell and Carriger, 1990; Brownell et al., 2006) and on behalf of others (Wu and Su, 2014). Moreover, framing social cognitive tasks as prosocial problems appears to facilitate performance (Matsui and Miura, 2008; Buttelmann et al., 2009), suggesting that prosocial behaviors are integrally entwined with the development of human social-cognition (see also Brownell et al., 2013b for a review). Given the automatic and pervasive role that mental state understanding plays in a wide variety of human interactions, and the central role prosocial behaviors play in human social success, it is plausible that the ability to represent others’ mental states accurately is a necessary prerequisite for early prosocial behavior.

One of the easiest, and most assured, ways of benefitting another involves intervening when they are faced with a negative experience. With this in mind, prosocial behaviors can be thought to require three components: (1) the ability to take the perspective of another person and recognize that they are having a problem; (2) the ability to determine the cause of that problem; and (3) the motivation to help them overcome the problem. Indeed, simply recognizing that someone is distressed is of little value if one is not willing to actually do something about it, nor is motivation helpful if you don’t know how to intervene. Together, the ability to successfully navigate each of these steps is necessary – but not alone sufficient – for the production of effective prosocial behavior; if an individual is unable to overcome any of these three challenges then a successful intervention is unlikely.

To be clear, the claim is not that *all* prosocial behaviors are *always* motivated by the direct perception of another’s negative state. Instead, the proposal is that the earliest instances of prosocial behaviors likely are, and that by considering the social cognitive constraints related to recognizing a negative state and identifying an appropriate intervention, we may gain better insight into how prosocial behaviors develop and change over early life. Adults are clearly motivated by imagined or implied distress and engage in prosocial behavior even in the absence of direct perception of a problem. At some point in development (potentially as early as the start of the second year, e.g., Vaish et al., 2009; Knudsen and Liszkowski, 2013; Warneken, 2013), humans can use imagined or inferred negative states as prosocial impetus. Without belittling the impressive developmental challenges that underlie the internalization of prosocial motivation, there is an important explanatory role for understanding how very young children come to recognize, interpret, and overcome the negative states that they directly perceive in others.

## CATEGORIZING PROSOCIAL BEHAVIOR

To reiterate, early prosocial behaviors rest on the ability to recognize that another is having a negative experience, the ability to determine what an appropriate response would entail, and finally, the motivation to intervene. With these constraints in mind, it is helpful to consider the types of negative states that individuals may need to recognize and respond to when engaging with others. Broadly considered, humans appear to experience three varieties of negatives states: *instrumental need*, where an individual has difficulty completing goal directed behavior; *unmet material desire,* in which the individual does not have access to a particular resource; and *emotional distress,* when an individual experiences a negatively arousing emotional state. Further, each of these negative states can be alleviated by a different variety of prosocial behavior namely, *helping* (e.g., retrieving an out of reach object; Warneken and Tomasello, 2006), *sharing* (e.g., giving up a limited resource, Hay, 1979; Brownell et al., 2009), and *comforting* (e.g., offering verbal or physical support; Vaish et al., 2009; Svetlova et al., 2010), respectively.

Because these three varieties of prosocial behavior are thought to rely on different initial social-cognitive assessments (i.e., goals, desires, and emotions), and the ability to represent these various mental states show unique patterns of development (e.g., Wellman and Woolley, 1990; Repacholi and Gopnik, 1997; Woodward, 1998; Wellman and Liu, 2004; Wellman et al., 2011), we should not necessarily predict consistency in the age of emergence, developmental trajectories, or supporting mechanisms for each variety of prosocial behavior. Looking to the existing literature on children’s social cognitive development, we find support for this position.

### INSTRUMENTAL NEED

#### Representing the problem

Helping requires the ability to accurately represent an instrumental need. Representing an instrumental need requires the ability to attribute an intended goal despite incomplete observations. Previous research suggests that within the first year of life infants can represent simple goal directed action (Woodward, 1998; Csibra et al., 1999), and shortly thereafter they can differentiate intentional from unintentional acts and recreate intended acts despite incomplete observations (Carpenter et al., 1998; Behne et al., 2005). For example, between 5 and 9 months, infants begin to construe others’ actions in terms of goals, not motions, showing greater interest in actors that change the target, as opposed to direction, of their reach (Woodward, 1998). By 8 months, infants identify and preferentially imitate intended behaviors, even when they are paired with accidental behaviors (Carpenter et al., 1998). Finally, by 9 months, infants prefer, and show more patience towards, individuals who fail to share because they are unable (and kept dropping the toy out of reach) as opposed to unwilling (and kept pulling the toy out of reach; Behne et al., 2005). Together, these studies demonstrate that between the end of the first year and start of the second year, infants are able to represent other’s behaviors in terms of their underlying goal structure and, despite observing incomplete actions, differentiate intended from unintended outcomes.

#### Representing the solution

In addition to being able to represent the goal structure underlying and organizing behavior, effective helping requires the ability to recognize effective interventions that support goal completion. An understanding of goals, and a preference for individuals associated with goal completion, appears to develop within the first year of life. For example, 8-month-olds expect individuals to display positive emotions following goal completion (Skerry and Spelke, 2014). By 2 years, infants display sympathetic nervous system arousal in response to incomplete goals, which is reduced after they witness the individual receive help, regardless of whether the help is self or other initiated (Hepach et al., 2012). Finally, when infants witness a character trying but failing to complete a goal, they prefer the character that was helpful (Hamlin et al., 2007) and expect others to share this preference (Kuhlmeier et al., 2003). And although these studies were not specifically intended to assess infants’ understanding of effective goal interventions, the only way infants could have made sense of the various interactions is by representing an initial goal (e.g., getting up the hill), representing the appropriate intervention (e.g., pushing to the top), and understanding that individuals are positively inclined towards completed goals.

Finally, utilizing a behavioral reenactment paradigm, Meltzoff (1995) provides the clearest evidence that by 18 months infants not only represent other’s actions as goal directed and prefer individuals and situations associated with completed goals, but also that they can represent and reproduce goals that they have not witnessed completed. Children watched as an experimenter tried but failed to complete a number of actions such as pulling apart a dumb bell or hanging a hoop on a post. The children were then given the opportunity to produce the actions themselves. Consistent with an ability to represent human action through the organizing lens of goals, the infants preferentially produced the actor’s intended outcome (e.g., pulled the barbells apart and hung the hoop) despite the fact they had never seen these goals completed, simply implied.

Together, it is clear from the extant literature that before the second birthday, children represent others’ actions in terms of underlying goals, recognize when and why goals may fail to be completed, and are highly motivated to see goals achieved. This suggests that within the first two years of life, children have developed the social cognitive skills required to support the recognition of instrumental need and produce helping behaviors.

### UNMET MATERIAL DESIRE

#### Representing the problem

Sharing, on the other hand, requires the ability and willingness to represent another’s unmet material desire. Typically, this involves recognizing and rectifying an unequal distribution of resources. In adults, allotments tend to be governed by the norm of fair distribution and associated with the “principle of equality,” which proposes that *ceteris paribus* goods should be divided equally among potential recipients, particularly when the primary goal of the interaction involves fostering and maintaining “enjoyable social relations” (Deutsch, 1975, p. 143). This tendency is well established in adults (e.g., Henrich et al., 2005; Baumard et al., 2013) and appears to emerge relatively early in development (e.g., Fehr et al., 2008; Sloane et al., 2012). Yet, unlike goal understanding, which has been extensively studied outside of the domain of prosocial behavior, the majority of the work that speaks to children’s understanding of resource inequality has been examined in relation to sharing behaviors.

Despite a long history of debate regarding whether children under the age of 5 are sensitive to unequal distributions of resources (e.g., Lane and Coon, 1972; Damon, 1975; Fehr et al., 2008), recent research utilizing a variety of converging implicit measures suggests that infants begin to recognize unequal distributions, and prefer equal distributions, early in their second year of life. Specifically, infants show greater attention to unfair (i.e., unequal) as opposed to fair (i.e., equal) distributions, suggesting that they expect resources to be divided fairly (e.g., Sloane et al., 2012). Indeed, multiple studies, conducted across a variety of labs, confirm this tendency (Geraci and Surian, 2011; Schmidt and Sommerville, 2011; Sommerville et al., 2013).

Critically, this preference for equal outcomes appears specific to social interactions. Infants do not show a similar pattern of looking when the recipient is inanimate, ruling out a low-level perceptual preference for equal amounts (Sloane et al., 2012). Moreover, consistent with the recognition that, in general, it is preferable to share items equally between recipients, infants prefer (based on reaching behavior) and expect others to prefer (based on looking time preferences) equal distributors (Geraci and Surian, 2011). Finally, consistent with the claim that representing an unmet material desire is uniquely important to the development of sharing behavior, infants’ sensitivity to unfair outcomes correlates with concurrent sharing (Schmidt and Sommerville, 2011) but not helping (Sommerville et al., 2013).

Although children under the age of 5 show mixed results articulating norms and expectations of fairness, when response demands are reduced and implicit measures (such as affective behavior) are used, children as young as 3 years of age recognize and respond negatively to unfair distributions of resources (LoBue et al., 2011). Specifically, children display clear negative emotions in response to unequal distributions and when prompted, identify such outcomes as “unfair” (especially when the participant is in the disadvantaged position). Together, this research suggests that the ability to represent, and negatively evaluate, unequal access to resources emerges over the course of the second year of development.

#### Representing the solution

Effectively alleviating material desire requires the ability to recognize an unequal distribution of resources, the motivation to see equality restored, and the ability to overcome an egocentric desire to monopolize resources. Although children can recognize unequal distributions of resources at least by 15 months, it is not clear that recognizing inequality is, in and of itself, sufficient to account for sharing behavior. Indeed, a compelling point raised by comparative researchers is that even when chimpanzees (and other non-human primates) can recognize an unfair offer, they are not necessarily motivated to act in order the change the situation (e.g., Brosnan, 2013). Moreover, even when children *do* act to change situations, it is not always clear whether their behaviors are directed at the alleviation of material desire *per se*, or are a manifestation of an impulse to engage socially (Tomasello et al., 2005).

When children are given the opportunity to divide resources between themselves and others, or select between predetermined divisions, there is a general trend towards fairer behavior with age. For example, when children are given the opportunity to divide resources on behalf of another, children as young as 3 work to ensure equal distributions (Olson and Spelke, 2008; Shaw and Olson, 2012). However, when children are making decisions that affect the self, an aversion to disadvantageous equality (i.e., rejecting offers that favor the other, e.g., 1 – self, 4 – other) emerges around 4 years of age, while opposition to advantageous inequality (i.e., rejecting offers that favor the self, e.g., 4 – self, 1 – other) emerges much later, between the ages of 6 and 8 years (Blake and McAuliffe, 2011).

Interestingly, despite having the ability to articulate the norm of fairness as young as 3, children do not always follow it. For example, Smith et al. (2013) found that children could report that they should distribute resources fairly and expected others to do so, yet when given the chance to divide resources, they showed a preference for self. Most amusingly, children seem well aware of their limits; though they knew they should share fairly, and expected others to do so, when asked what they would do when given the opportunity to share, participants correctly predicted that they would behave selfishly.

Finally, a recent study that employed both experimental control and a naturalistic social context demonstrated an increase in the frequency and spontaneity of early sharing behavior between 18 and 24 months (Brownell et al., 2013a). Specifically, participants were given access to food and toys in the presence of an adult experimenter who had none. Unlike many of the studies examining resource distribution, the participants were not explicitly instructed to divide the resources. Instead, the adult playmate expressed her desire using a series of progressively more explicit cues. Eighteen-month-olds were willing to share but often only after the experimenter made her desire explicit. In contrast, by 24 months, participants shared spontaneously, often immediately, and typically more generously than at 18 months. Moreover, consistent with an important role for understanding another’s desire in the emergence of sharing behavior, sharing was positively associated with understanding of self and ownership, and negatively associated with self-focused behaviors (e.g., ignoring the experimenter) and hypothesis testing (e.g., staring at the experimenter).

In sum, children recognize the importance of equal outcomes within the first two years of life; however, the tendency to spontaneously act to resolve these issues shows protracted development. Moreover, there are a number of situational factors that influence whether children will apply their recognition of unequal outcomes to remedy an unfair situation. For example, sharing in children under the age of 3 can be increased when others make their desire explicit (e.g., Brownell et al., 2009, 2013a; Dunfield et al., 2011), the cost of sharing is low (e.g., Thompson et al., 1997; Moore, 2009), or the recipient is familiar (Rheingold et al., 1976; Hay, 1979; Hay and Murray, 1982). Together these findings providing further support for the proposal that recognizing unmet material desire (i.e., an unequal outcome) alone is not sufficient for effective sharing, particularly when the solution is unclear, or motivation is weak.

### EMOTIONAL DISTRESS

#### Representing the problem

Comforting requires the ability to represent another’s negative emotional state. Effectively representing another’s emotional distress requires the ability to differentiate and identify the various emotional experiences of others. From the earliest days of life, infants respond to other’s distress with distress of their own (e.g., Sagi and Hoffman, 1976). Yet, despite the integral role that emotional contagion is thought to play in the development of sympathy and comforting behavior (see Hoffman, 1982; Preston and De Waal, 2002; Decety and Meyer, 2008 for reviews), it is not sufficient to support effective other-oriented responses to distress. Instead, it is the ability to identify both another’s negative emotional state, and the cause, that likely supports effective comforting behavior.

Researchers have demonstrated the foundations of the ability to identify negative emotional states in early infancy. As early as 3 months of age, infants can differentiate the facial expressions of happiness from surprise and anger, and by 7 months, infants can additionally represent fear, sadness, and interest (Grossmann, 2010). Developing in concert with the ability to discriminate between various emotional expressions is the ability to represent the equivalency of various emotional cues. For example, around 7 months of age, infants begin to recognize conflicting emotional expressions (e.g., when a sad face is paired with a happy voice) and preferentially attend to pairings that are emotionally consistent (e.g., a happy face paired with a happy voice; Walker-Andrews and Dickson, 1997). Together, these results suggest that within the first year of life infants differentiate positive and negative emotions, with differentiation between varieties of negative affect developing shortly thereafter.

Consistent with many developmental accomplishments, children’s emotion recognition appears to vary depending on the task demands. Although infants can differentiate varieties of emotional expressions and recognize cross-modal congruence in implicit tasks within the first year of life, it is not until almost 3 years of age that they show a limited ability to discuss a restricted range of emotions (Denham and Couchoud, 1990). The development of children’s ability to explicitly label others’ emotions mirrors the developmental progression observed with implicit measures. Specifically, while children as young as 2 years can label happiness, it takes an additional year or two before they can reliably identify negative emotions such as anger, fear, and sadness (Denham and Couchoud, 1990; Widen and Russell, 2003). As a whole, these studies suggest that while some of the necessary emotional understanding is in place in the first year of life (i.e., emotional discrimination and expectations of consistency), many of the requisite skills (i.e., explicitly identifying the particular type of distress) do not emerge until later toddlerhood.

#### Representing the solution

Simply recognizing another’s negative emotions is not sufficient to support mature comforting behavior. Being able to identify the cause of another’s emotional state is critically important for understanding and intervening on their behalf (e.g., Saarni et al., 2006). Indeed, the social, emotional, and cognitive developments that children experience over the first year of life – which allow them to progress from mirroring another’s negative emotion to representing the negative state and understanding a cause and solution – have long been thought to be an integral part of prosocial development (Hoffman, 1982, 2000).

Children’s understanding of the idiosyncratic nature of emotions emerges in the second year of life. For example, though 14-month-olds overgeneralize their personal preferences, 18-month-olds recognized that individuals might differ in their emotional experiences (Repacholi and Gopnik, 1997). Relatedly, children as young as 2, understand that situational factors influence both emotions and behaviors (Wellman and Woolley, 1990). Then, by three children can make accurate predictions regarding the types of situations that lead to happiness and between 4 and 5 start making accurate predictions about situations that lead to anger, fear, or surprise (Denham and Couchoud, 1990; Widen and Russell, 2003).

Finally, children not only recognize situations that lead to various emotions, but also the contextual appropriateness of emotional expressions. As early as 18 months infants have expectations regarding likely emotional reactions, engaging in more checking behavior and less concerned attention when witnessing unjustified as opposed to justified distress (i.e., distress following positive versus negative outcomes respectively; Chiarella and Poulin-DuBois, 2013). Further, by 3 years of age, children will show concern, offer assistance, and even check on an individual who has displayed justifiable distress, while largely ignoring an individual whose distress is unjustified (Hepach et al., 2013). It appears as though the appropriateness of the emotion plays an important role in early distress intervention.

Thus, although infants can recognize consistency in emotional expressions within the first year of life, the ability to represent, track, and respond appropriately to the person-specific idiosyncratic nature of emotions takes much longer to develop. Indeed, consistent with Hoffman’s early theoretical account, the ability to represent another’s emotional distress alone is not sufficient for effective comforting interactions. Instead, it is likely that effective other-oriented comforting should emerge over the course of the second to fourth years and capitalize on a growing understanding of the unique, diverse, and situationally constrained nature of others’ emotional experiences.

## PROSOCIAL BEHAVIOR AS HELPING, SHARING, AND COMFORTING SUBTYPES

**FIGURE 1:**
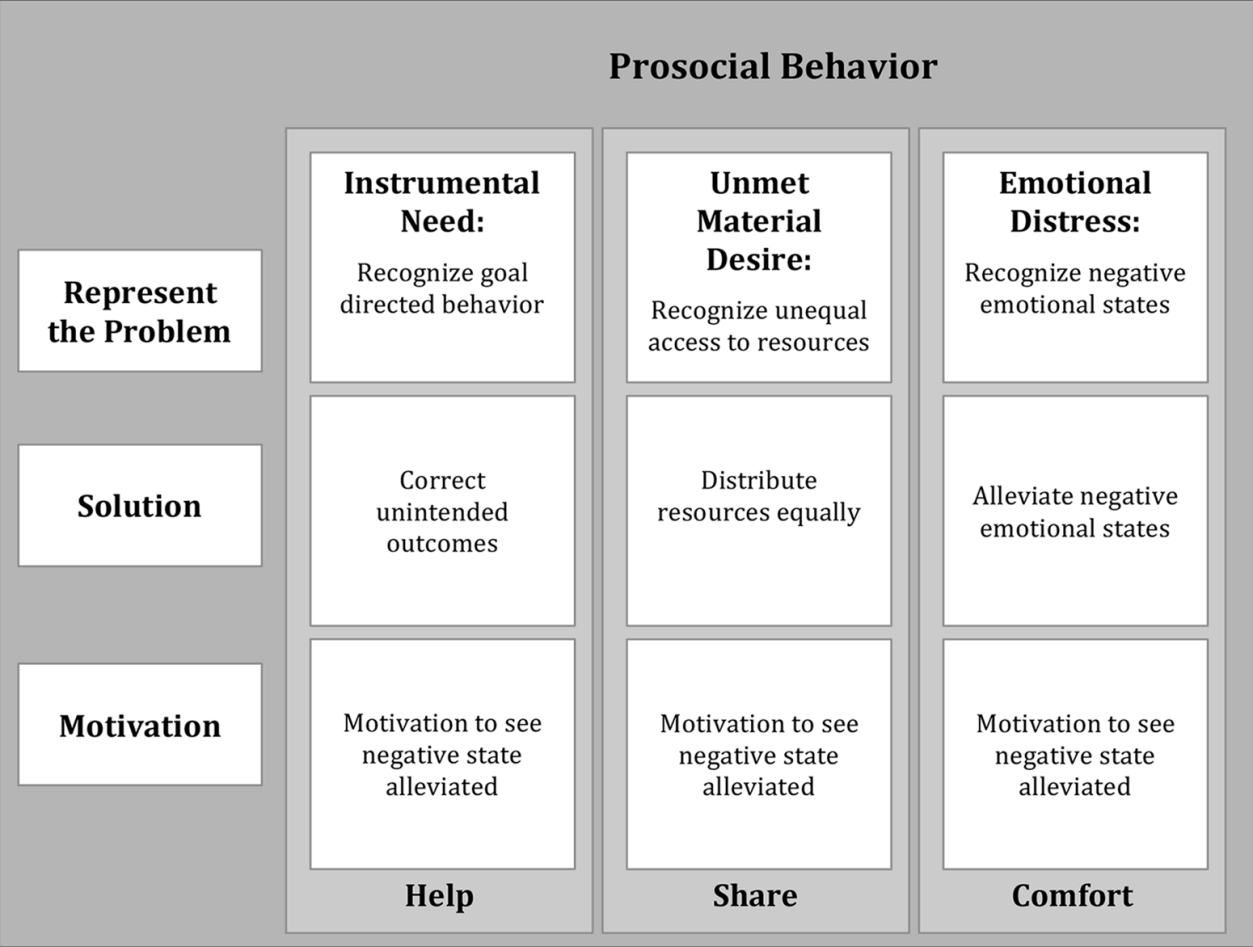
**Categorization of prosocial behavior based on the varieties of negative state the child must identify and overcome.** An effective intervention will only occur when all three components can be successfully resolved. Different varieties of prosocial behavior show independent developmental trajectories because of the unique social cognitive demands.

To summarize, this categorization (**Figure 1**) proposes that within the general domain of prosocial behavior there are three more specific varieties of behavior that individuals engage in, namely helping, sharing, and comforting. Moreover, each of these three varieties of behavior is elicited by a unique negative state: instrumental need, material desire, and emotional distress, respectively. Because the successful production of an effective prosocial intervention relies largely on the ability to recognize the presence of a negative state and determine the cause of the negative state, this categorization allows us to make a number of predictions: (1) Prosocial behavior should be more likely to occur when a negative state is present than when it is absent. (2) Different varieties of prosocial behavior should emerge at different ages and develop along different trajectories based on the underlying social-cognitive constraints. (3) Finally, individual difference factors should affect the various form of prosocial behavior differently depending on how they influence the underlying constraints. In the following sections, I will briefly present a selection of relevant research that speak to these predictions and support the utility of this categorization.

### RESPONDING TO NEGATIVE STATES

One of the major contributions of this categorization is that it predicts that other-oriented acts, especially ones produced early in life, are more likely to occur when the child is able to represent another’s negative state. Recent research provides strong support for this proposal. Warneken and Tomasello (2006) developed a novel experimental paradigm that clearly demonstrates that by 18 months, children will intervene helpfully when they observe an unknown adult in need of help. Unlike much previous research, this study included an elegant control condition that allowed for a systematic investigation of the role of need in the production of prosocial behavior. In experimental trials, the children saw the experimenter genuinely trying and failing to complete a goal, whereas in control trials the children observed the same behaviors manipulated to obscure the experimenter’s need. Across a variety of tasks, 18-month-olds showed a sensitivity to need, helping only in situations where the experimenter was actually having difficulty completing an intended goal.

Capitalizing on this powerful experimental design, more recent studies have examined infants’ ability to respond to all three of the proposed negative states (Dunfield et al., 2011). Specifically, infants were presented with both an experimental and control trial for instrumental need, unmet material desire, and emotional distress. In experimental trials the negative state was clearly present. In control trials however, the participants observed identical surface behavior with the negative state obscured. Consistent with the proposal that prosocial behavior relies on the ability to represent the negative states of another, both 18- and 24-month-olds were found to help and share when instrumental need and material desire were present (experimental trials), but not in highly similar situations where the negative states were absent (control condition). Even in the case of emotional distress, in which children failed to differentiate between the experimental and control conditions, it was not because they inappropriately offered comfort in the absence of a distress cue; instead, they simply failed to demonstrate any prosocial behavior.

Consistent with an important role for representing negative states in the production of prosocial behavior, young children are more likely to act prosocially when the appropriate intervention is made obvious, or the specific negative state and appropriate intervention is made explicit (e.g., Brownell et al., 2009, 2013a; Svetlova et al., 2010; Dunfield and Kuhlmeier, 2013). For example, Svetlova et al. (2010) gave 18- and 30-month-olds the opportunity to respond to multiple prosocial “requests” in which the children could alleviate the experimenter’s distress by offering her various objects; over the course of each trial the experimenter exhibited up to eight increasingly specific cues that eventually highlighted the particular need and the appropriate intervention. Two patterns of results were particularly compelling: (1) 30-month-olds required less explicit cuing than 18-month-olds, and (2) children were more likely to assist when the experimenter’s difficulty was instrumental as opposed to emotional. Together these results support the proposal that early in development the ability to represent another’s negative state limits when and how children produce prosocial behavior.

Moreover, consistent with an important role for negative state understanding in the production of effective prosocial behavior, 3-year-olds will override an experimenter’s specific request (e.g., for a cup that the child knows is broken) in order to provide more effective solutions (e.g., for another cup that was not requested but functional; Martin and Olson, 2013). Taken together, there is mounting support for the proposal that differences in the age and conditions under which children’s early prosocial behaviors develop may be accounted for, at least in part, by the developing ability to represent accurately the negative mental states of others.

Finally, though early prosocial behaviors are often observed in response to negative states, it is not the case that all prosocial behaviors are always motivated by the direct perception of difficulty. For example, while 14- and 18-month olds are more likely to help an experimenter who notices, and reaches for a dropped object (Warneken and Tomasello, 2006, 2007), by 30-months children helpfully retrieve dropped objects that were unnoticed by the experimenter (Warneken, 2013), suggesting that children quickly internalize situations that lead to instrumental need.

Moreover, as predicted by the categorization, some negative states are unrelated to the production of a prosocial intervention. Specifically, consistent with the claim that helping is a specific response to an instrumental need, the addition of negative affect does not increase helping behavior (Newton et al., 2014). Yet, the ability to take another’s affective perspective, even in the absence of displayed negative affect, influences children’s motivation to share following the observation of a clearly unmet material desire (Vaish et al., 2009). Further, in cases where a goal has been demonstrated and an impediment to goal completion is made clear, children as young as 18 months can communicate helpfully to aid an experimenter in avoiding a negative outcome (i.e., *before* the problem occurs, Knudsen and Liszkowski, 2013).

Together, these studies support the important fit between the representation of a particular negative state and the ability to produce an appropriate prosocial intervention. Yet they also highlight an important role for future research in better understanding when and how these evaluations get internalized. Moreover, they suggest more research is required to understand how individuals come to triage between negative states to determine the core issue that needs to be addressed in order to appropriately and effectively aid another.

### AGE OF EMERGENCE AND DEVELOPMENTAL TRAJECTORIES

Another prediction of this categorization is that varieties of prosocial behavior should emerge at different ages and develop along distinct developmental trajectories due to the fact they rely on different mental state attributions, which develop along different trajectories. Though previous research has suggested that prosocial behavior emerges between the first and second birthday and increases in frequency and complexity as the child ages (e.g., Hoffman, 1982; Zahn-Waxler et al., 1992; Hay, 1994; Eisenberg et al., 2006), it is not clear that this claim applies equally to all varieties of prosocial responses.

Looking to the existing literature reviewed above, children should be able to respond to instrumental need prior to unmet material desire and emotional distress, both of which will show more variability and context dependence due to the later emerging social cognitive supports. Consistent with this prediction, helping appears to be one of the earliest emerging forms of prosocial behavior, beginning shortly after the child’s first birthday (Warneken and Tomasello, 2007) and showing rapid development over the first half of the second year (Warneken and Tomasello, 2006). Sharing appears to emerge later in the second year increasing in frequency and spontaneity between 18 and 24 months (Brownell et al., 2013a), supported by a clear articulation of desire (Brownell et al., 2009, 2013a), and a reduction of inhibitory demands (e.g., Olson and Spelke, 2008; Smith et al., 2013). Finally, as expected, children’s ability to alleviate another’s emotional distress with other-oriented comforting behavior emerges last (Dunfield and Kuhlmeier, 2013) and is preceded by concerned attention (Spinrad and Stifter, 2006), and facilitated by clarifying the appropriate intervention (Svetlova et al., 2010).

We see the same pattern of production when the three negative states are presented within-subject, suggesting this is not a methodological artifact but instead a characteristic of early other-oriented behaviors (Dunfield et al., 2011; Dunfield and Kuhlmeier, 2013). Further, tasks that use subsets of prosocial behavior converge, showing that relative to helping, comforting emerges later (Radke-Yarrow et al., 1976) and sharing appear less frequent (Radke-Yarrow et al., 1976; Grusec, 1991; Eisenberg, 2005).

Together, the existing literature supports the claim that early prosocial behaviors show unique patterns of emergence as a function of the specific negative state they address. Further, these studies are consistent with the position that the ability to understand others’ negative mental states influences the age at which children can intervene prosocially on behalf of others. Indeed, children are more likely to assist others when the negative state is made clear and the appropriate intervention is simple, suggesting an important facilitatory role for mental-state understanding in the development of children’s prosocial responses.

A closely related prediction is that the production of various forms of other-oriented behavior should not necessarily correlate. Dunfield and Kuhlmeier (2013) gave 2-, 3-, and 4-year-olds the opportunity to respond to four instances of instrumental need, unmet material desire, and emotional distress. Because the children were given the opportunity to respond to multiple instances of multiple varieties of each of the three negative states, it was possible to examine correlations both within and across tasks. Consistent with the proposed utility of the present categorization, participants reliably responded to a particular negative state, while responses *across* negative states remained uncorrelated. Thompson and Newton (2013), find consistent behavioral results and similarly suggest that differences in the production of varieties of prosocial behavior may relate to the unique underlying social-cognitive constraints. Finally, in support of these interpretations, it appears that helping and comforting are associated with distinct, dissociable neural correlates (sharing was not examined; Paulus et al., 2013).

Taken together, there is mounting support for the proposal that helping, sharing, and comforting reflect unique varieties of prosocial behaviors with distinct ages of onset (Dunfield et al., 2011), unique uncorrelated developmental trajectories (Dunfield and Kuhlmeier, 2013; however, see Thompson and Newton, 2013 for an alternative explanation), and distinct underlying neurophysiological supports (Paulus et al., 2013). Each of these findings are consistent with the utility in dividing the general domain of prosocial behavior into three more specific varieties based on the unique mental state they respond to.

### VARIABILITY IN DEVELOPMENT

The third prediction is that individual differences will not necessarily influence each variety of prosocial behavior equally. A number of individual difference factors have been found to affect the production of prosocial behavior as a whole (for comprehensive reviews see Eisenberg et al., 2006, in press). However, because these studies were not intended to examine whether different prosocial behavior are differentially affected by individual difference factors, it is not possible to determine whether these factors have a similar influence on all proposed varieties of prosocial behaviors or instead exert their influences selectively. If the proposed categorization based on negative state attribution is going to be useful in organizing the examination of prosocial behavior, then it should help predict and explain differences in the production of prosocial behavior across individuals. Specifically, an individual difference factor should only affect the production of a particular prosocial behavior if it influences the ability to represent, or the motivation to resolve, a particular negative state. In this section I will demonstrate how variations in social cognition, emotion processing, socialization, and culture assert different influences on the three proposed varieties of prosocial behavior.

#### Autism

One factor that that may affect the ability to represent, and motivation to assist in overcoming, another’s negative state is a diagnosis of autism spectrum disorder (ASD). Children with ASD develop social cognitive abilities along an atypical trajectory (e.g., Charman et al., 1998; Dyck et al., 2001) and receive less reinforcement from shared social interactions (Dawson et al., 2004). This suggest that children with autism may have a harder time recognizing and interpreting each of the three negative states and possess less motivation to see another’s negative state overcome.

The few studies that do exist examining prosocial behaviors in children with autism found that while children with ASD engage in simple helping and sharing (Liebal et al., 2008), they are unlikely to respond to observations of distress (e.g., Sigman et al., 1992; Travis et al., 2001; Hobson et al., 2009). When given the opportunity to respond to all three varieties of prosocial behavior in a controlled experimental paradigm (see Section “Methods” in Dunfield et al., 2011), children with ASD responded to material desire and emotional distress, but surprisingly, not instrumental need (Dunfield et al., 2012). Although these children were much older (the mean age was 46 months) than Dunfield et al.’s (2011) sample, the overall pattern of results was opposite, with comforting and sharing preceding helping, suggesting that the unique suite of social-cognitive abilities and deficits that characterize ASD do indeed differentially affect the three varieties of prosocial behavior. However, it is not currently possible to determine if these effects are a function of difficulty representing the displayed negative state, or limited motivation to interact, future research will be required to determine at which stage in the prosocial process children with autism are experiencing difficulty.

#### Attachment security

A second individual difference factor that has been observed to differentially affect the ability to represent the various negatives states is attachment security. Attachment security refers to the extent to which individuals believe that they can depend on others to have their needs met, and their expectations regarding others’ tendencies to seek and accept comfort (e.g., Bowlby, 1982). Securely attached individuals generally see other people as reliable sources of support, whereas insecurely attached individuals see others as unreliable sources of potential pain (e.g., Dykas and Cassidy, 2011). And although attachment security has been generally associated with the production of empathic behaviors across the lifespan (Mikulincer et al., 2001; Mikulincer and Shaver, 2005; Mikulincer et al., 2005; Diamond et al., 2012), it is possible that it does not affect the ability to represent all three varieties of negative states equally (Johnson et al., 2013).

Specifically, though infants appear to have universal expectations regarding instrumental interventions (e.g., Kuhlmeier et al., 2003; Hamlin et al., 2007), their expectations regarding emotionally distressing situations appears to differ based on attachment security (e.g., Johnson et al., 2007, 2010). When university undergraduates are given the opportunity to describe social interactions where the specific negative state is ambiguous, securely attached individuals identify both instrumental need and social-emotional distress with equal ease, while insecurely attached individuals preferentially avoid discussing social-emotional distress (Dunfield, 2012; Johnson et al., 2013). Attachment security appears to represent a second domain of individual difference that exerts a differential effect on the ability to represent the various negative states. Future research will need to examine whether and how these different representations affect the production of the three varieties of prosocial behavior.

#### Socialization

While the focus of this paper has largely been the importance of considering underlying, species universal, social cognitive mechanisms that differentiate varieties of prosocial behaviors, socialization plays an integral role in the emergence and production of prosocial behavior (e.g., Rheingold, 1982; Hay, 1994). Styles of caregiving, play, and discipline have all been found to influence children’s tendency to respond sensitively and appropriately to the observation of another’s distress (for a complete review of the socialization of prosocial behavior, see Hastings et al., 2007; Eisenberg et al., in press). Particularly relevant to the current proposal is the idea that there are at least three pathways through which socialization can influence the production of prosocial behavior (e.g., Brownell et al., 2013c). Specifically, socialization could affect the production of prosocial behavior by increasing motivation (e.g., Dunn, 2008), supporting self-regulatory skills (e.g., Eisenberg, 2000; Spinrad and Stifter, 2006), or supporting the development of underlying social cognitive abilities (e.g., Denham et al., 1994; Ensor et al., 2011).

While it is clear that socialization is fundamentally important to supporting the production of prosocial behavior, it is not clear that all types of socialization are equally effective in encouraging all varieties of prosocial behavior. For example, a recent study (Pettygrove et al., 2013) investigated the relation between parental socialization and prosocial behavior by giving 18- and 30-month olds the opportunity to help, share, and comfort in response to increasingly explicit cues to the experimenter’s negative state. Additionally, parental socialization techniques were coded while the parent and child interacted in a different but related task. The researchers replicated previous findings regarding the unique, uncorrelated production of prosocial behavior in early development. Moreover, they demonstrate that varieties of prosocial behaviors were differentially affected by varieties of socialization techniques, finding that the most effective socialization techniques were ones that targeted the child’s particular developmental need.

However, socialization influences do not always show distinct relations with varieties of prosocial behaviors. For example, parents who frequently elicited emotion talk from their children tended to have children who helped *and* shared more quickly and frequently than children who engaged in less emotion discussion (Brownell et al., 2013c). Looking to the three components that are proposed to support effective prosocial behavior, it is possible that factors that influence the ability to represent the underlying negative state and solution may require different socializing influences (e.g., Pettygrove et al., 2013) than factors affecting motivation to act on behalf of others (e.g., Brownell et al., 2013c). Specifically, though socialization undoubtedly plays an important role in supporting when and how children act on behalf of others, considering the unique constraints that underlie the varieties of prosocial behavior may lead to more nuanced understanding of the variety of ways that socialization exerts its influence. This categorization of prosocial behavior, based on the unique and dissociable social-cognitive constraints that underlie other-oriented acts, could aid in better understanding when, how, and why, varieties of prosocial are differentially influenced by socialization.

#### Culture

Although it is well established that humans universally engage in prosocial behaviors (e.g., Henrich et al., 2005), there appears to be culture-specific variability in the developmental trajectories (Rochat et al., 2009; Callaghan et al., 2011), frequency (Graves and Graves, 1983; Williams, 1991), and social cognitive influences (Kärtner et al., 2010) underlying varieties of prosocial behavior (for more comprehensive reviews see Drummond et al., in press; Hammond et al., in press). Specifically, cultures seem to vary in the types of prosocial behaviors they value, beliefs about who is deserving of prosocial behavior, and the manner in which social-cognitive abilities support the production of prosocial behavior (e.g., de Guzman et al., 2008; Knafo et al., 2009).

There is relatively little systematic cross-cultural research examining the production of multiple varieties of prosocial behavior, particularly in early childhood, but the studies that do exist suggest that some components of prosocial development are shared across cultures, while others vary. For example, though mothers from Peru, India, and China all report that their infants begin helping between 14 and 17 months, they identified different types of helping behavior (Callaghan et al., 2011). Specifically, Peruvian and Indian children tended to only help with household tasks, while Canadian children also engaged in self-helping behaviors such as dressing and putting away toys. Mothers also reported different motivations underlying helping; Peruvian mothers saw helping as a natural behavior, Indian mothers saw it as reflection of their child’s understanding of need, whereas Canadian mothers saw it as a function of social learning. Yet, despite these differential self-reports, by 18 months children from all three cultures identified instrumental need and preferentially helped when need was present.

When sharing behavior is examined across a number of diverse cultural contexts (i.e., rich and poor urban environments, small-scale traditional and rural communities; Rochat et al., 2009), the general trend of 3-year-olds engaging in relatively self-interested behavior that becomes increasingly other-oriented by 5 is replicated. Moreover, the results hinted at a universal association between the development of social cognition and increasingly generous behavior. However, despite considerable similarity, there are important differences in the level of self-interest the youngest children started with and magnitude of the developmental differences across the various cultures tested.

Finally, when given an opportunity to respond to an experimenter’s emotional distress, 19-month-olds in Berlin and Delhi were equally likely to recognize and respond to an experimenter’s negative emotional state (Kärtner et al., 2010). Yet, despite responding similarly to distress cues, the two cultures differed in the socialization goals they emphasized and the role of social cognitive development in the production of pseudo-comforting behavior. Specifically, mothers from Delhi tended to emphasize more relational socialization goals than mothers from Berlin whereas, mirror self-recognition predicted distress and comforting behavior in Berlin but not Delhi. Together these results suggest that there may be a number of distinct developmental routes that lead to similar behavioral outcomes.

Though the tendency to produce prosocial behaviors is a human universal, there is considerable cultural variability in the form and development of other-oriented acts. Culture may exert its influence on the development of prosocial behavior by selectively emphasizing particular values and then affording differential socialization opportunities (e.g., Keller, 2007). Moreover, depending on the cultural context of development, it is possible that the same developmental outcome (i.e., effective other-oriented behavior) may emerge along different pathways. To that end, research that specifically examines varieties of prosocial behavior and their associated social-cognitive supports will be in a better position to understand the nuanced development of these fundamental social behaviors.

Taken together, the reviewed lines of research suggest that individual difference factors do not necessarily exert the same influence on all varieties of prosocial behavior. Specifically, it is important to consider the fit between the social-cognitive or motivational effects of a particular individual difference variable and the demands of a particular variety of prosocial behavior when predicting how the two will interact. While exciting and suggestive, this line of inquiry is still in its infancy. An important direction for future research will involve a more systematic examination of how various individual differences affect the representations and motivations underlying the three varieties of negative states and the extent to which these differences affect the types and frequencies of prosocial behaviors that children produce.

## SUMMARY

The goal of this paper was to address some of the inconsistencies in our understanding of the early emergence and development of prosocial behavior by considering the social-cognitive constraints that underlie the ability to act on behalf of others. This social-cognitive categorization of prosocial behavior proposes that within the general domain of prosocial behavior, other-oriented actions can be categorized into three distinct types namely: helping, sharing, and comforting. Each of these varieties of prosocial behavior relies on the recognition of, and response to, a distinct negative state namely: instrumental need, unmet material desire, and emotional distress, respectively. By distinguishing between these three negative states we are in a better position to identify the *distinct* social cognitive abilities that support each type of prosocial behavior. Importantly, by doing so we can begin to better understand the unique ages of onset, uncorrelated patterns of production, and distinct patterns of individual differences that are currently challenging our understanding of the earliest instances of these fundamental human behaviors.

## Conflict of Interest Statement

The author declares that the research was conducted in the absence of any commercial or financial relationships that could be construed as a potential conflict of interest.
